# Improving the sorting efficiency of maize haploid kernels using an NMR-based method with oil content double thresholds

**DOI:** 10.1186/s13007-020-00703-4

**Published:** 2021-01-06

**Authors:** Yanzhi Qu, Zonghua Liu, Yazhou Zhang, Jiwei Yang, Haochuan Li

**Affiliations:** College of Agronomy, National Key Laboratory of Wheat and Maize Crop Science, Collaborative Innovation Center of Henan Grain Crops, Henan Agricultural University, Zhengzhou, China

**Keywords:** Maize, Haploid, Single kernel weight, Oil content, NMR, Double-threshold method

## Abstract

**Background:**

Maize haploid breeding technology can be used to rapidly develop homozygous lines, significantly shorten the breeding cycle and improve breeding efficiency. Rapid and accurate sorting haploid kernels is a prerequisite for the large-scale application of this technology. At present, the automatic haploid sorting based on nuclear magnetic resonance (NMR) using a single threshold method has been realized. However, embryo-aborted (EmA) kernels are usually produced during in vivo haploid induction, and both haploids and EmA kernels have lower oil content and are separated together using a single threshold method based on NMR. This leads to a higher haploid false discrimination rate (FDR) and requires secondary manual sorting to select the haploid kernels from the mixtures, which increases the sorting cost and decreases the haploid sorting efficiency. In order to improve the correct discrimination rate (CDR) in sorting haploids, a method to distinguish EmA kernels is required.

**Results:**

Single kernel weight and oil content were measured for the diploid, haploid, and EmA kernels derived from three maize hybrids and nine inbred lines by in vivo induction. The results showed that the distribution of oil content showed defined boundaries between the three types of kernels, while the single kernel weight didn't. According to the distribution of oil content in the three types of kernels, a double-threshold method was proposed to distinguish the embryo-aborted kernels, haploid and diploid kernels based on NMR and their oil content. The double thresholds were set based on the minimum oil content of diploid kernels and the maximum content of EmA kernels as the upper and lower boundary values, respectively. The CDR of EmA kernels in different maize materials was > 97.8%, and the average FDR was reduced by 27.9 percent.

**Conclusions:**

The oil content is an appropriate indicator to discriminate diploid, haploid and EmA kernels. An oil content double-threshold method based on NMR was first developed in this study to identify the three types of kernels. This methodology could reduce the FDR of haploids and improve the sorting efficiency of automated sorting system. Thus, this technique represents a potentially efficient method for haploid sorting and provides a reference for the process of automated sorting of haploid kernels with high efficiency using NMR.

## Background

Maize haploid breeding can significantly shorten the breeding cycle and improve breeding efficiency, and it has become a popular and routine technology used in maize breeding programs [1]. At present, haploids are mainly produced by in vivo induction in maize breeding. Fast and accurate sorting of haploids is a prerequisite for the use of haploid breeding technology and is also a key point in haploid engineering breeding. Early haploid sorting methods can be performed at various seedling and plant growth stages. At the seedling stage, the traits of radicle length, coleoptile length, and the number of lateral seminal roots of haploid seedlings are significantly different from diploids [2]. In addition, the number of chromosomes, stomata and chloroplasts and the DNA content of haploids are significantly lower than those of diploids [3, 4], so flow cytometry and microscopy are usually used to identify haploid and diploid individuals. However, these methods are carried out after seed germination or even at the seedling stage, the processes are complicated, and the efficiency is low. Haploid identification can also be conducted at the plant growth stage based on morphological characteristics; haploid plants have upright and narrow leaves and grow slowly, and plant height and ear position are significantly lower than those of diploid plants [5–7]. Nevertheless, the above methods are only feasible in the field or greenhouse; additionally, these methods delay haploid identification and lead to resource waste, so they are generally not used in maize haploid breeding.

In maize haploid breeding, it is ideal to discriminate haploids at the kernel stage. Presently, haploid kernels are mainly sorted by hand based on the dominant anthocyanin synthesis gene *R1-nj* (Navajo), which imparts a purple color to both the embryo and endosperm in double fertilized (diploid) kernels but only in the endosperm of haploid kernels, allowing haploids to be identified and screened by their colorless embryos [8–10]. However, manual sorting is not efficient for haploid kernels with unclear color expression in the embryo and endosperm [11, 12], and results in high labor costs and high time-consumption. In order to improve the efficiency of haploid sorting, the first automatic sorting equipment was developed by means of computer vision. This system was based on the *R1-nj* color marker expressed in the embryo to identify haploid and diploid kernels, the correct discrimination rate (CDR) of sorting was ~ 80% [13, 14]. Nevertheless, haploid kernels cannot be identified by imaging when the embryo side is facing away from the light source. For this reason, the rugged slope module was appended, and a high-speed camera was used to acquire multiple pieces of information from each kernel, which resulted in the CDR of 95% [15], but the haploids could not be sorted in flint corn and tropical germplasms, where the expression of the *R1-nj* (Navajo) marker is usually inhibited [16–18]. Moreover, deep convolutional networks and convolutional neural networks based on *R1-nj* color, texture and morphology were used to discriminate haploids with an accuracy of more than 94% [19, 20], but embryo-aborted (EmA) kernels were not identified. Furthermore, fluorescence tags, as another novel indicator, have been used to detect haploids in immature embryos. Diploid embryos show a fluorescence signal after pollination using a fluorescent inducer with the yellow fluorescent protein (YFP) signal as the male, while haploid embryos do not [21–23]. The fluorescence signal of YFP is usually weak in mature embryos, so it is limited in application. The spectral method has also been used for screening of haploid kernels, after obtaining the spectral features by near infrared spectroscopy (NIR) [24–27], diffuse transmission [28], kernel locality preserving projection [29], and hyperspectral imaging technology [30, 31]. Machine learning algorithms or deep belief networks were used to construct a haploid selection model to analyze the NIR spectral data, and the haploid sorting accuracy reached over 90% [32, 33]. A quantitative analysis method based on the spectral features of the oil content of kernels was developed to sort haploids, and the haploid sorting accuracy was above 90% [34, 35]. The spectral method provides a new concept in haploid sorting; however, repeated modeling will take a long time, and no equipment has yet been developed based on the spectra for the automatic sorting of maize haploid kernels.

The xenia effect is a common phenomenon in hybrid seed formation in maize, and the effect of xenia on the oil content in maize kernels is especially obvious because it can significantly increase the oil content of hybrid seeds when high oil lines are used as pollen donors [36]. China Agricultural University first reported that the oil xenia effect could be an indicator to discriminate haploids from hybrid kernels by their oil content, and the first high oil inducer line (CAUHOI) was successfully developed [37–39]. The xenia effect on oil content can lead to a significant difference in oil content between haploid (3.86%) and diploid (5.26%) kernels [36]. According to this principle, an automated haploid sorting system was developed based on nuclear magnetic resonance (NMR), and the CDR reached more than 92% [40, 41]. The feasibility of sorting haploid kernels using NMR was further confirmed [42] and shown to be a simple and reliable method to identify haploid kernels from large quantities of hybrid kernels, greatly improving the efficiency of haploid sorting [43]. Recent researches on sorting haploids based on NMR spectrum and manifold learning showed that the CDR reached as high as 98 and 90% for haploids induced by high-oil and conventional inducers, respectively [44]. Although the haploid CDR has been improved to some extent, the EmA kernels have not been sorted with this level of success. At present, oil content analysis is usually employed with a single threshold to discriminate haploid kernels using NMR. However, EmA kernels are produced along with haploid and diploid kernels on the same ears during in vivo haploid induction [45–47]. There is a highly positive correlation between the haploid induction rate (HIR) and the embryo abortion rate (EmAR) [47, 48]. The haploid and EmA kernels, both of which have lower oil content than diploid kernels, are usually mixed together during the process of haploid sorting by NMR. This leads to a higher haploid false discrimination rate (FDR), and manual secondary sorting is then needed to select the haploid kernels from the mixtures, which increases the sorting cost and decreases the haploid sorting efficiency. Therefore, the proper identification of EmA kernels is an important step in automated sorting systems based on NMR. However, little information is available about sorting EmA kernels. The objectives of this study were to (1) analyze the embryo abortion rate (EmAR) in different parental materials by in vivo haploid induction; (2) measure the single kernel weight and oil content of EmA, haploid, and diploid kernels; and (3) propose and verify the effectiveness of a double-threshold method for sorting haploids to reduce the FDR in maize haploid kernel sorting.

## Results

### Variations in the embryo abortion rate

EmA kernels appear in different crosses during the process of haploid induction. In the crosses using three hybrids as females and four different inducers as males, the EmARs of the three hybrids were 4.14, 2.20, and 1.47%, respectively. The hybrid G1 (4F1/Zheng58) had the highest EmAR at 4.14% when the M1 (CAUHOI) inducer was used as the male. The three hybrids had the lowest EmARs of 2.31, 1.14, and 0.71% when the M2 (CHOI2) inducer was used as the male. The EmARs of the different inbred lines differed after in vivo induction, and ranged from 4.32–11.93%; the inbred line G6 (Lx9801) had the highest EmAR at 11.93%, while the inbred line G12 (L217) had the lowest EmAR at 4.32%. The average EmAR and HIR were 4.64% and 7.77%, respectively (Table 1). These results indicated that the identification of EmA kernels is important during haploid kernel sorting.

**Table 1:** EmAR in different maize hybrids following pollination with four different inducers

Female	Male	Diploid (k)	Haploid (k)	EmA (k)	EmAR (%)	HIR (%)
G1	M4	429	11	16	3.51	2.5
	M3	327	18	18	4.96	5.22
	M2	304	38	21	5.78	11.11
	M1	820	25	20	2.31	2.96
G2	M4	676	32	15	2.07	4.52
	M3	1207	89	23	1.74	6.86
	M2	767	84	34	3.84	9.87
	M1	2116	49	25	1.14	2.26
G3	M4	1027	55	10	0.92	5.08
	M3	1370	110	43	2.82	7.43
	M2	1335	117	21	1.43	8.06
	M1	3778	121	28	0.71	3.1
G4	M2	863	84	55	5.74	8.83
G5	M2	411	41	46	9.2	9.03
G6	M2	413	30	60	11.93	6.77
G7	M2	396	56	32	6.53	12.23
G8	M2	299	57	20	5.22	15.7
G9	M2	385	43	21	4.63	9.93
G10	M2	408	32	42	8.61	7.17
G11	M2	336	40	42	10.02	10.61
G12	M2	511	85	27	4.32	13.91
Average of all materials		866	58	29	4.64	7.77

*EmAR* embryo abortion rate

## Comparison of single kernel weight and oil content in the different kernel types

**Fig. 1:**
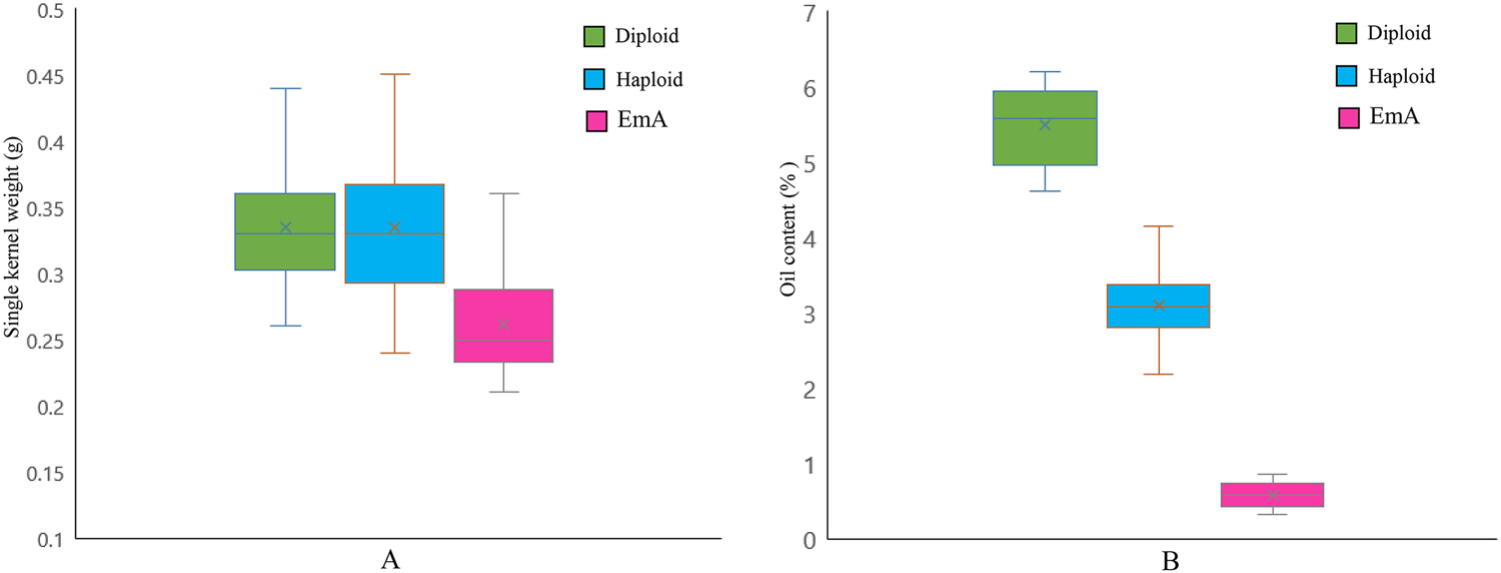
Boxplots showing combined single kernel weight (**a**) and oil content (**b**)

The single kernel weight of diploid, haploid, and EmA kernels from the hybrid G1 (4F1/Zheng58) were 0.44 g, 0.45 g, and 0.36 g, respectively, and these were the highest weight of each kernel type in the experiment. The three kernel types from the inbred line G10 (Dan 598) had the lowest weight; the single kernel weight of diploid, haploid, and EmA kernels were 0.26 g, 0.26 g, and 0.21 g, respectively. The single kernel weight of diploid and haploid kernels was similar; the mean single kernel weight for EmA kernels was 0.26 g, which was lower than the mean weight for single diploid and haploid kernels (Table 2**)**. The results of statistical analyses (Student’s *t* test) showed that the differences in single kernel weight were highly significant between EmA and diploid kernels (t = 8.01, *p* < 0.01) and between EmA and haploid kernels (t = 7.10, *p* < 0.01), but there was no significant difference between diploid and haploid kernels (t = 0, *p* > 0.05). Therefore, haploid sorting cannot be achieved based on single kernel weight (Fig. 1). For the kernel oil content, we found that the oil content of diploid kernels from the hybrid G1 (4F1/Zheng58) and the inbred lines G9 (P138) and G11 (Zheng22) were 6.04, 6.20, and 5.93%, respectively. These were the highest values for diploid kernels, while the oil content of haploid kernels from inbred line G6 (Lx9801) was the lowest at 2.19%, and the average oil contents of all haploid except for G8 and EmA kernels were < 3.5% and 1%, respectively. Student’s *t*-test showed that the difference in oil content was highly significant in comparisons of EmA and diploid kernels (t = 30.88, *p* < 0.01) and EmA and haploid kernels (t = 15.50, *p* < 0.01) and between diploid and haploid kernels (t = 16.71, *p* < 0.01). Therefore, the oil content can be used to identify haploid, diploid, and EmA kernels with a high degree of confidence.

**Table 2:** Analysis of single kernel weight and oil content in diploid, haploid, and EmA kernels

Material	Single kernel weight (g)			Oil content (%)		
	Diploid	Haploid	EmA	Diploid	Haploid	EmA
G1	0.44 ± 0.03	0.45 ± 0.04	0.36 ± 0.02	6.04 ± 0.67	3.34 ± 0.40	0.63 ± 0.37
G2	0.33 ± 0.04	0.34 ± 0.04	0.30 ± 0.04	5.56 ± 0.79	3.17 ± 0.57	0.65 ± 0.31
G3	0.42 ± 0.04	0.42 ± 0.04	0.29 ± 0.04	5.59 ± 0.68	3.43 ± 0.46	0.44 ± 0.35
G4	0.27 ± 0.04	0.27 ± 0.05	0.25 ± 0.04	5.6 ± 0.65	3.37 ± 0.46	0.66 ± 0.45
G5	0.36 ± 0.03	0.36 ± 0.03	0.28 ± 0.05	5.47 ± 0.80	2.82 ± 0.62	0.53 ± 0.42
G6	0.31 ± 0.05	0.30 ± 0.04	0.25 ± 0.04	5.39 ± 0.66	2.19 ± 0.61	0.81 ± 0.39
G7	0.33 ± 0.06	0.33 ± 0.06	0.25 ± 0.05	4.72 ± 0.53	2.80 ± 0.43	0.41 ± 0.49
G8	0.36 ± 0.06	0.37 ± 0.05	0.24 ± 0.05	5.93 ± 0.67	4.14 ± 0.18	0.52 ± 0.36
G9	0.33 ± 0.05	0.32 ± 0.04	0.25 ± 0.04	6.20 ± 0.71	3.27 ± 0.44	0.33 ± 0.33
G10	0.26 ± 0.05	0.24 ± 0.04	0.21 ± 0.03	4.81 ± 0.68	2.96 ± 0.48	0.36 ± 0.38
G11	0.31 ± 0.06	0.33 ± 0.06	0.23 ± 0.03	5.93 ± 0.84	2.97 ± 0.57	0.86 ± 0.26
G12	0.30 ± 0.05	0.29 ± 0.05	0.23 ± 0.04	4.61 ± 0.59	2.77 ± 0.47	0.77 ± 0.46

### Distribution of the single kernel weight and oil content in the different kernel types

**Fig. 2:**
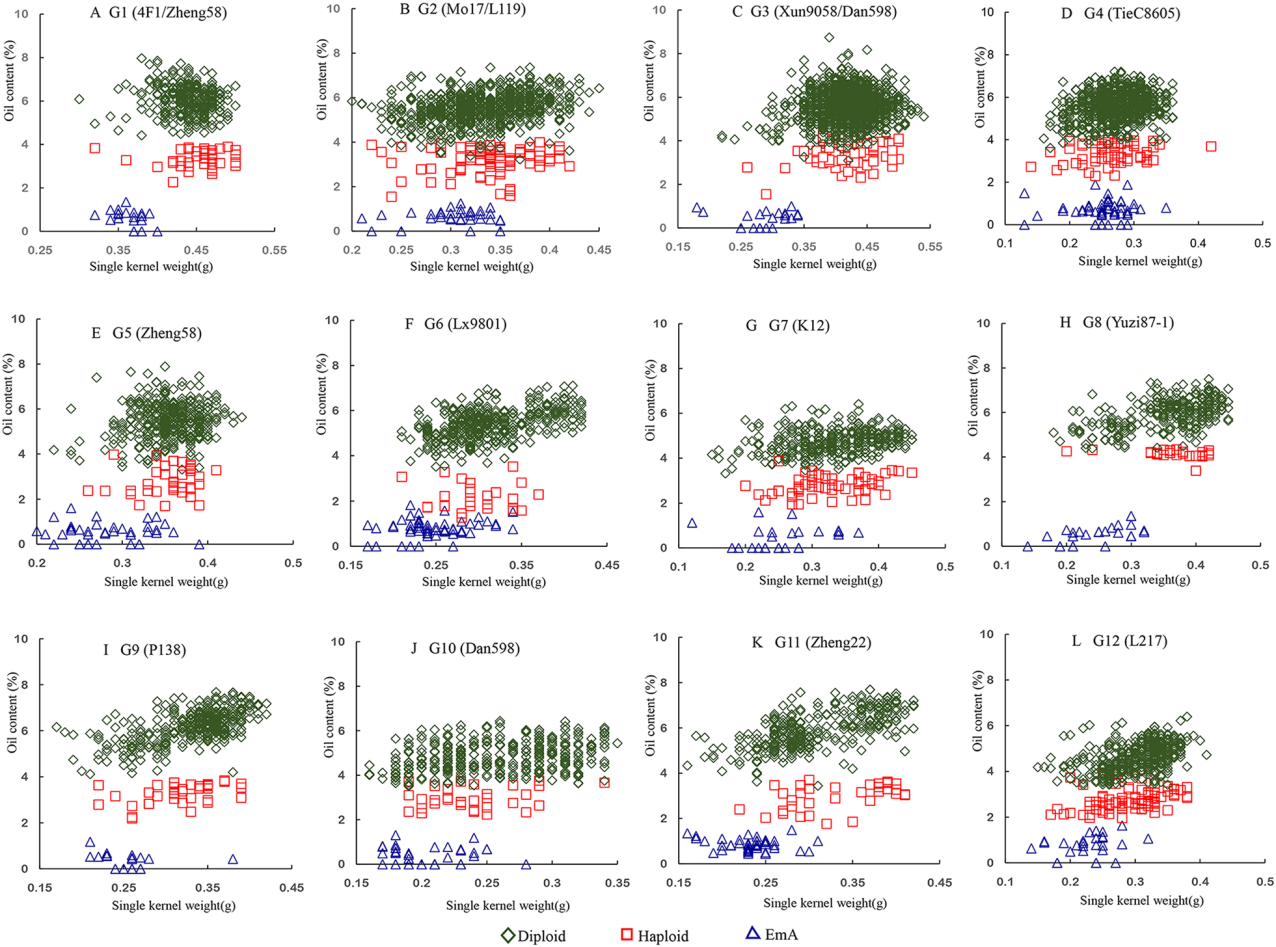
Distribution of oil content and single kernel weight for the three different kernel types

The oil content of diploid, haploid, and EmA kernels showed a clear distribution range in each experimental material (Fig. 2). In all panels, the diploid kernels had the highest oil content, the haploids had the middle oil content, the EmA kernels had the lowest oil content, and the boundaries between the three were clear (Fig. 2). Therefore, it was easy to identify the three different kernel types on the basis of their oil content. Single kernel weight appeared to show a continuous distribution (Fig. 2), and there was no obvious discontinuous distribution among the three kernel types because there were overlaps between any two types of kernels. Therefore, the oil content is an appropriate indicator to distinguish diploid, haploid, and EmA kernels during in vivo haploid induction using high oil inducers. The minimum oil content of diploid kernels was set as the upper limit, and the maximum oil content of EmA kernels was set as the lower limit; this double threshold for the oil content could be used to discriminate diploid, haploid, and EmA kernels in the NMR automated sorting system. Any kernels with oil contents above the upper limit or below the lower limit would be considered as non-haploids and sorted together, while kernels with oil content between the upper and lower limits would be regarded as haploids.

### Haploid sorting using NMR and a double-threshold for the oil content

#### Analysis of the CDR for EmA kernels

Based on the distribution of the oil content for EmA and haploid kernels (Fig. 2), the oil content of EmA kernels was < 2.0%, while it was > 2.0% for haploid kernels (Fig. 2). Therefore, an oil content of 2.0% was set as the lower threshold to test the sorting of EmA kernels, and the results showed that the average CDR for EmA kernels was 97.8%. Among the 12 maize hybrids and inbred lines used in this study, the lowest CDR for EmA kernels was 78% for G6 (Lx9801), followed by 95% for G5 (Zheng58), while the kernels from the remaining 10 materials were sorted completely with a 100% CDR for EmA kernels (Table 3).

**Table 3:** The CDR_EmA_ of the 12 different maize materials using the double-threshold method

Materials	Frequency					Average
	1	2	3	4	5	
G1	100.0	100.0	100.0	100.0	100.0	100.0
G2	100.0	100.0	100.0	100.0	100.0	100.0
G3	100.0	100.0	100.0	100.0	100.0	100.0
G4	100.0	100.0	100.0	100.0	100.0	100.0
G5	95.0	90.0	90.0	100.0	100.0	95.0
G6	80.0	80.0	70.0	75.0	85.0	78.0
G7	100.0	100.0	100.0	100.0	100.0	100.0
G8	100.0	100.0	100.0	100.0	100.0	100.0
G9	100.0	100.0	100.0	100.0	100.0	100.0
G10	100.0	100.0	100.0	100.0	100.0	100.0
G11	100.0	100.0	100.0	100.0	100.0	100.0
G12	100.0	100.0	100.0	100.0	100.0	100.0
Average	97.9	97.5	96.7	97.9	98.8	97.8

#### Analysis of CDR_H_, CRR_D_ and FDR

In order to determine the upper limit of the double-threshold method, five different oil contents of 3.0, 3.5, 4.0, 4.5, and 5.0% were set to test their effects on the correct discrimination rate of haploids (CDR_H_) and the correct rejection rate of diploids (CRR_D_), which are two important standards for judging the efficiency of haploid sorting. The CDR_H_ increased with an increasing upper limit value; when the upper limit threshold for the oil content was set at 5.0%, the average CDR_H_ was 93.9%, and nine out of 12 materials reached 100% (Table 4). The CDR_H_ was lower, only 32.5%, at an oil content threshold of 3.0%, and the CDR was < 50% for most of the materials. However, the trend for CRR_D_ was the opposite of that for CDR_H_; at the 3.0% oil content, all hybrids and inbred lines had CRR_D_ values of 100%, while the lowest CRR_D_ at 5.0% oil content was only 82.3%. A higher oil content threshold was better for sorting haploid kernels, and a lower threshold was better for sorting diploid kernels. The CDR_H_ and CRR_D_ would allow haploid kernels to be sorted correctly, and analysis of the CDR_H_ and CRR_D_ combination showed that the average rate could be > 90% at a threshold oil content of 4.5%. Thus, the upper limit of the double threshold for the different maize materials is between 3.5 and 4.5% (Table 4). For a single threshold, the average FDR was > 50% at five different oil content thresholds, and the FDR was the highest (65.1%) at a 3% oil content threshold. For the double thresholds, the FDR was 44.6% lower than that of the single-threshold method, and the lowest rate was only 0.9% at a 3.0% oil content threshold. Thus, using a double-threshold method can significantly reduce the FDR in contrast to using a single threshold (Table 4).

**Table 4:** The CDR_H_, CRR_D_, and FDR of different materials using the double-threshold method

**Index**	**Threshold**	**Female Materials**												**Mean**
		**G1**	**G2**	**G3**	**G4**	**G5**	**G6**	**G7**	**G8**	**G9**	**G10**	**G11**	**G12**	
**CDR**_**H**_	**5.0%**	100.0	100.0	100.0	93.3	93.3	100.0	100.0	40.0	100.0	100.0	100.0	100.0	93.9
	**4.5%**	100.0	90.0	83.3	90.0	100.0	100.0	100.0	20.0	73.3	100.0	83.3	100.0	86.7
	**4.0%**	93.3	66.7	70.0	53.3	90.0	100.0	96.7	3.3	50.0	83.3	76.7	100.0	73.6
	**3.5%**	50.0	30.0	40.0	23.3	76.7	93.3	90.0	0.0	26.7	43.3	60.0	80.0	51.1
	**3.0%**	23.3	13.3	16.7	16.7	50.0	80.0	63.3	0.0	3.3	20.0	40.0	63.3	32.5
**CRR**_**D**_	**5.0%**	91.3	88.7	94.7	93.3	67.3	86.7	36.7	100.0	100.0	60.0	98.0	70.7	82.3
	**4.5%**	100.0	99.3	99.3	98.7	88.0	99.3	71.3	100.0	100.0	88.7	100.0	89.3	94.5
	**4.0%**	100.0	100.0	99.3	100.0	90.7	100.0	96.7	100.0	100.0	97.3	100.0	97.3	98.4
	**3.5%**	100.0	100.0	100.0	100.0	96.7	100.0	100.0	100.0	100.0	100.0	100.0	100.0	99.7
	**3.0%**	100.0	100.0	100.0	100.0	100.0	100.0	100.0	100.0	100.0	100.0	100.0	100.0	100.0
**Average of CDR**_**H**_** and CRR**_**D**_	**5.0%**	95.7	94.3	97.3	93.3	80.3	93.3	68.3	70.0	100.0	80.0	99.0	85.3	88.1
	**4.5%**	100.0	94.7	91.3	94.3	94.0	99.7	85.7	60.0	86.7	94.3	91.7	94.7	90.6
	**4.0%**	96.7	83.3	84.7	76.7	90.3	100.0	96.7	51.7	75.0	90.3	88.3	98.7	86.0
	**3.5%**	75.0	65.0	70.0	61.7	86.7	96.7	95.0	50.0	63.3	71.7	80.0	90.0	75.4
	**3.0%**	61.7	56.7	58.3	58.3	75.0	90.0	81.7	50.0	51.7	60.0	70.0	81.7	66.3
**FDR; single threshold**	**5.0%**	43.4	55.2	48.3	51.7	71.1	57.1	79.3	62.5	40.0	72.7	43.4	68.1	57.7
	**4.5%**	40.0	43.8	45.7	44.9	55.9	41.2	67.7	76.9	47.6	55.2	44.4	54.5	51.5
	**4.0%**	41.7	50.0	50.0	55.6	55.7	40.0	46.3	95.2	57.1	49.0	46.5	44.4	52.6
	**3.5%**	57.1	69.0	62.5	74.1	52.1	41.7	42.6	100.0	71.4	60.6	52.6	45.5	60.8
	**3.0%**	74.1	83.3	80.0	80.0	57.1	45.5	51.3	100.0	95.2	100.0	62.5	51.3	65.1
**FDR; double threshold**	**5.0%**	30.2	36.2	21.1	26.3	64.1	44.4	76.0	0.0	0.0	66.7	9.1	59.5	36.1
	**4.5%**	0.0	3.6	3.8	6.9	40.0	14.3	58.9	0.0	0.0	36.2	0.0	34.8	16.5
	**4.0%**	0.0	0.0	4.5	0.0	37.2	16.7	14.7	0.0	0.0	13.8	0.0	11.8	8.2
	**3.5%**	0.0	0.0	0.0	0.0	17.9	15.2	0.0	0.0	0.0	0.0	0.0	0.0	2.8
	**3.0%**	0.0	0.0	0.0	0.0	0.0	11.1	0.0	0.0	0.0	0.0	0.0	0.0	0.9

*CDR*_*H*_ correct discrimination rate of haploid kernels, *CRR*_*D*_ correct rejection rate of diploid kernels, *FDR* false discrimination rate

### Comparison between single- and double-threshold methods

The FDR, another important indicator to evaluate haploid sorting efficiency, is defined as the percent of non-haploid kernels in the haploid kernel group. CDR_H_ and CRR_D_ were the same for both the single- and double-threshold methods, but the FDR of the double-threshold method was reduced by 27.9% compared to that when using a single-threshold method. The minimum FDR for the hybrid G1 (4F1/Zheng58) and inbred line G9 (P138) was 40% (Table 5) using a single threshold because the haploid and EmA kernels were mixed together, and five of the 12 maize inbred lines and hybrids had FDRs > 50%. Compared with a single threshold, using a double-threshold can effectively reduce the FDR; kernels from G1 (4F1/Zheng58) and G9 (P138) showed the greatest reduction, with an FDR for both materials of 0. The FDR also declined in the other materials with the use of a double-threshold (Table 5). This indicates that the double-threshold method can effectively reduce the FDR of haploids by correct identification of EmA kernels.

**Table 5:** Comparisons of the FDRs of different materials using single- and double-threshold methods

Materials	Single threshold			Double threshold		
	CDRH	FDR	CRRD	CDR_H_	FDR	CRRD
G1	100.0	40.0	100.0	100.0	0.0	100.0
G2	100.0	55.2	88.7	100.0	36.2	88.7
G3	100.0	48.3	94.7	100.0	21.1	94.7
G4	93.3	51.7	93.3	93.3	26.3	93.3
G5	100.0	57.1	88.0	100.0	40.0	88.0
G6	100.0	45.5	99.3	100.0	14.3	99.3
G7	96.7	46.3	96.7	96.7	14.7	96.7
G8	100.0	56.5	87.3	100.0	38.8	87.3
G9	100.0	40.0	100.0	100.0	0.0	100.0
G10	100.0	55.2	88.7	100.0	36.2	88.7
G11	100.0	43.4	98.0	100.0	9.1	98.0
G12	100.0	44.4	97.3	100.0	11.8	97.3
Average	99.2	48.6	94.3	99.2	20.7	94.3

*CDR*_*H*_ correct discrimination rate of haploid kernels, *CRR*_*D*_ correct rejection rate of diploid kernels, *FDR* false discrimination rate

## Discussion

In previous studies, the *R1-nj* color marker was usually used for automated sorting of maize haploid kernels. The principle behind this method is based on a computer vision system to discriminate haploid and diploid kernels by obtaining images of each kernel, and the CDR of haploids was ~ 80% [13, 14]. This color marker was also used to discriminate haploid kernels by artificial methods. However, the expression of this color marker is usually suppressed in maize kernels with the *C-I* gene; thus, it is not feasible to use this marker in some maize inbred lines or hybrids that carry inhibitors of *R1-nj* expression. China Agricultural University first reported that the xenia effect could be an indicator to identify haploid kernels [36]; the effects of oil mass, seed weight, and oil content on the sorting of haploid and diploid kernels were analyzed, and oil content was found to be optimal for classification [42]. In the automated NMR system based on oil content, the CDR for haploid kernels was 94% [41]. EmA kernels are a common phenomenon in in vivo haploid induction [21, 47, 49], and EmA kernels with lower oil contents are usually mixed with haploid kernels when a single threshold for oil content is used in sorting. In the present study, the percent of EmA to haploid kernels in maize hybrids and inbred lines were 53.6% and 87.4%, respectively. This implies that > 50.0% of the sorted kernels will be EmA kernels, requiring a second manual sorting, which increases the cost of sorting. Therefore, the CDR of haploid and EmA kernels from diploid kernels is an important part of automated haploid sorting. To this end, single kernel weight and oil content were measured for EmA, haploid, and diploid kernels during haploid induction. The results showed that a single kernel weight alone is not suitable for identifying the three types of kernels because there is no significant difference between diploid and haploid kernel weights, and there are overlaps between the weights of any two groups of kernels. However, the oil content showed an obvious difference between the diploid (5.5%), haploid (3.1%), and EmA (0.61%) kernels, so it could be used as an indicator. Based on this, a double threshold method based on evaluation of the oil content is first proposed for haploid kernel sorting in the present study; we found that this method reduced the haploid FDR by 27.9%, which improved the accuracy of haploid sorting and advanced haploid breeding technology in practice.

**Fig. 3:**
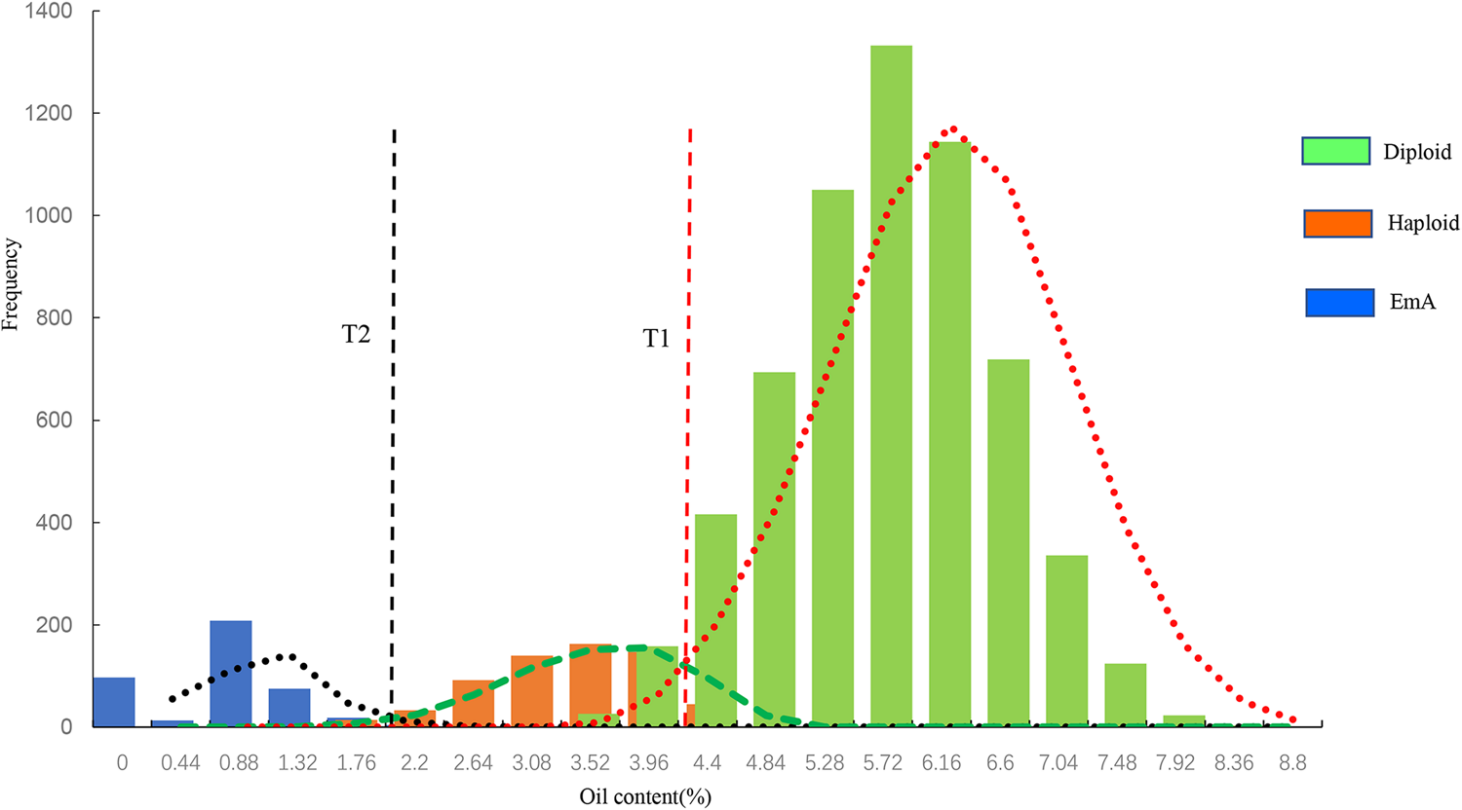
Distribution of oil contents for haploid, diploid, and EmA kernels. Dashed vertical lines labeled T1 and T2 indicate the upper and lower oil content thresholds for haploid kernels, respectively

There is a certain degree of overlap in the oil content between haploid and diploid kernels [41, 43], which leads to an increase in the haploid FDR. Therefore, the determination of threshold T1 is important, and previous studies have reported the determination of threshold T1 using the Bayesian classifier or the least squares error method [11, 50]. In our study, the average rate for the combination of CRR_D_ and CDR_H_ was > 90% as a standard for setting the upper limit thresholds. Threshold T1 for the different maize materials was 3.5–4.5%. However, EmA kernels could not be screened with a single threshold method. To sort EmA kernels, we had to also set a lower limit threshold, T2. There were obvious distinctions between the range of oil contents in EmA, haploid, and diploid kernels (Fig. 1b), and the oil content of EmA kernels was usually < 2.0% (Figs. 2, 3). The oil contents of diploid and haploid kernels were > 2.0%, which indicated that an oil content of 2% can be set as the lower limit, T2, to screen for EmA kernels. The kernels with oil contents between T1 and T2 were regarded as haploid, and kernels in which the oil content was < T2 and > T1 were regarded as EmA and diploid kernels, respectively, and were sorted together. Therefore, rapid and accurate sorting of haploid kernels can be realized by setting the appropriate oil content thresholds using the double-threshold method.

Initially, an oil content threshold was set to sort haploids using NMR, which neglected the existence of EmA kernels. Due to the lower oil content of EmA kernels, they were mistakenly recognized as haploid kernels during sorting, which resulted in a higher FDR of haploid discrimination. Although the phenotype of EmA kernels is easy to determine [51, 52], secondary sorting is needed, which increases the cost of sorting haploid kernels. Therefore, it was necessary to improve the haploid FDR. At present, there is no information about EmA kernel discrimination based on NMR. Based on the results from the single threshold method, we proposed the double-threshold method to sort haploid kernels, where in seeds produced by high oil inducers would be divided into two groups: haploid kernels group and a mixture of diploid and EmA kernels. By implementing the double-threshold sorting method, the CDR for EmA kernels (CDR_EmA_) reached 97.8%. The effect of sorting haploid kernels using a double-threshold was evaluated at five different oil content levels for the upper limit, and the haploid FDR was reduced by 44.6% compared with that of the single threshold method.

The maize haploid sorting system based on NMR using a double-threshold can save sorting costs in practice. Presently, the sorting system using the single threshold has a sorting speed of 4 s/kernel and can sort more than 20,000 kernels/day (24-h working time/day), it takes only one person less than 1 h to add kernel samples into the sample hopper, and the total cost is ¥ 15 yuan/day. Less than 3000 kernels/day are sorted by hand for each person, which will cost ¥ 120 yuan/day (8 h of working time a day). The automated sorting system can sort the same number of kernels as approximately 7 people sorting by hand, and the cost of manual sorting is ¥ 840 yuan/day. Therefore, the automated sorting system can reduced costs by ¥ 825 yuan per day compared to manual sorting. The double-threshold method can eliminate the step of manual secondary sorting and save ¥ 120 yuan/day compared to the single-threshold method and ¥ 945 yuan/day compared to manual sorting, which indicates that the double-threshold method can significantly reduce the cost of sorting haploid kernels.

Furthermore, another advantage of the double-threshold method is that it can reduce the haploid FDR and reduce the workload required for manual secondary sorting of EmA kernels from haploids. Furthermore, the automated haploid sorting system using NMR was based on the xenia effect of the oil content, which results in a difference in oil contents between diploid and haploid kernels. However, the xenia effect on the oil content depends on the genetic backgrounds of the male and female parents [53]. Lambert et al. used nine hybrids as females and pollinated them with both a high-oil and a normal-oil male to compare the effects of the two pollinators. They found that the oil contents of four normal-oil hybrids were 6.0–7.0% after pollination with a high oil pollinator, which was an increase of 1.7% in oil content in these same hybrids pollinated with the normal-oil male [54]. The oil content of hybrid seeds increases with increasing oil content of the male parent, and the influence of the male parent is greater than that of the female parent [55]. Thus, an effective method would be to select inducers with high seed oil contents and a stronger xenia effect, which will increase the difference in oil content between haploid and diploid seeds and reduce the overlap between them, effectively preventing diploid kernels from mixing with the haploids and further reducing the haploid FDR.

## Conclusions

The automated sorting of maize haploid kernels using NMR is a promising way to replace human labor. In order to discriminate between the diploid, haploid, and EmA kernels during haploid induction, single kernel weight and oil content were measured for the three types of kernels, and the results showed that the oil content was the better indicator in the haploid sorting system. Using a double-threshold for the oil content to sort haploid kernels was first proposed in the present study. The average CDR_*EmA*_ was > 97.8%, and the FDR was reduced by 27.9% with the double-threshold method compared to that with the single-threshold method. An oil content of 2.0% as the lower limit in the double-threshold method was suitable for most donors, and the mean overlap in the oil content between the haploid and diploid kernels can be used as the upper limit as a compromise. Our results verified the feasibility of discriminating between haploid, diploid, and EmA kernels with the double-threshold method, which will improve the haploid kernel sorting efficiency and reduce the cost of haploid identification.

## Materials and methods

### Experimental materials

**Fig. 4:**
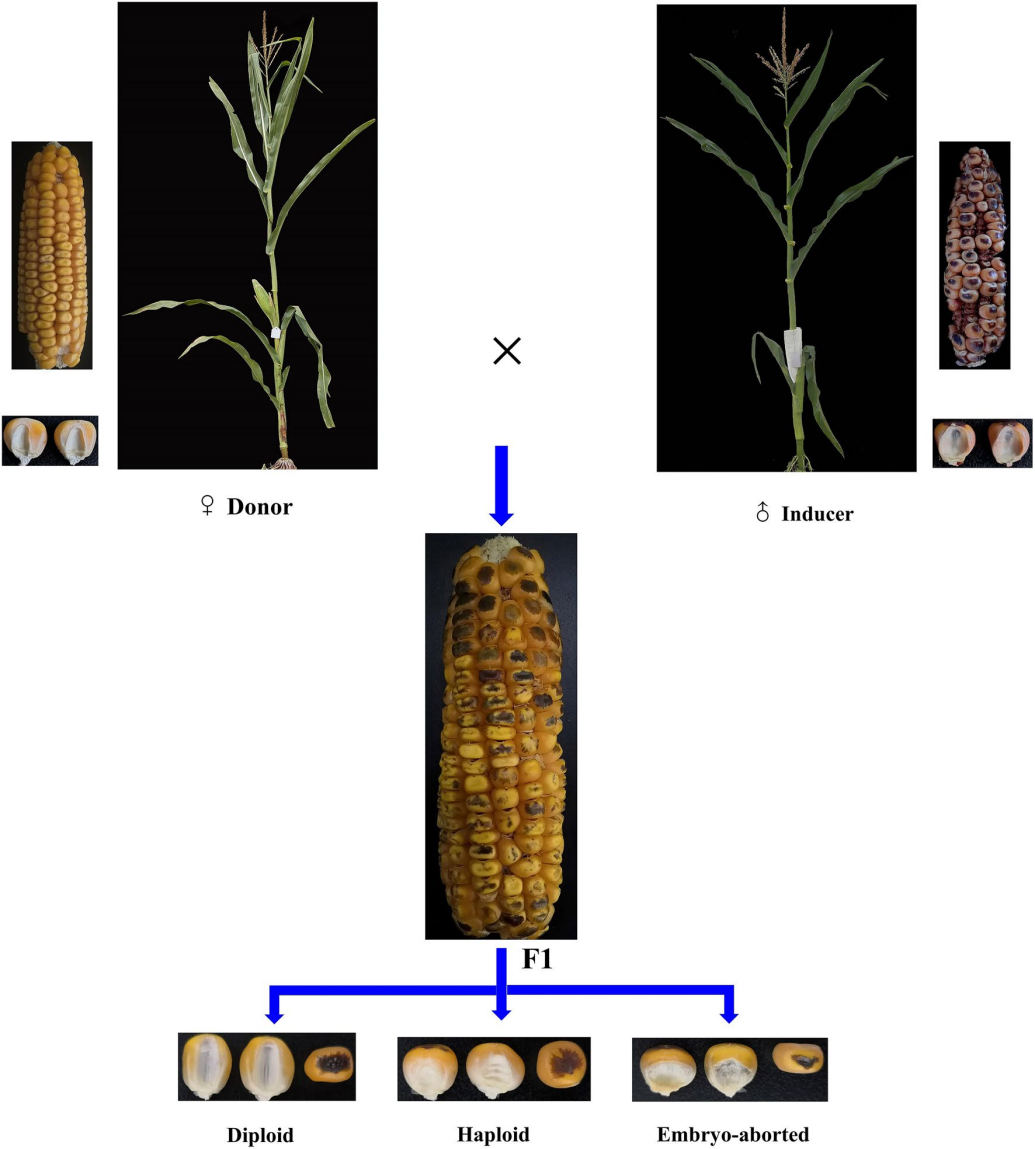
The process of maternal haploid induction by in vivo induction

Three maize inducer inbred lines, CAUHOI, CHOI2, YHI-1, and the inducer hybrid CAUHOI/CHOI2, were used as the male parents in this study. The haploid induction rates (HIRs) of CAUHOI and CHOI2 are ~ 2.0% and ~ 7%, respectively [38, 56], and both inducers were developed at China Agricultural University. The maize inducer YHI-1 with a HIR > 10% was developed at Henan Agricultural University [47, 57]. Three maize hybrids, 4F1/Zheng58, Mo17/L119A, and Xun9058/Dan598, were used as females in crosses with each inducer, and nine inbred lines (TieC8605-2, Zheng58; Lx9801, K12, Dan598, Zheng22, Yuzi87-1, P138, and L217) were selected for use as females to cross with CHOI2 (Table 6). Crosses were performed (Fig. 4) at the Hainan Experimental Station of Henan Agricultural University (18°21 N, 109°10E) during the winter of 2017. Plants were sown in single rows that were 4 m long with a 0.60 m distance between the rows and 20 plants per row. The female donors were detasseled before flowering. Standard agronomic practices in maize production such as irrigation, fertilization, and weeding were used during the entire growth period.

**Table 6:** Sources of male and female maize hybrids and inbred lines used in this study

No	Parent	Name	Germplasm	Type of material
M1	Male	CAUHOI	Inducer	Inbred line with high oil
M2	Male	CHOI2	Inducer	Inbred line with high oil
M3	Male	YHI-1	Inducer	Common inbred line
M4	Male	CAUHOI/CHOI2	Inducer	Hybrid with high oil
G1	Female	4F1/Zheng 58	Lancaster/Reid	Hybrid
G2	Female	Mo17/L119A	Lancaster/Tangsipingtou	Hybrid
G3	Female	Xun9058/Dan598	Reid/Lvdahonggu	Hybrid
G4	Female	Tie C8605	Reid	Inbred line
G5	Female	Zheng58	Reid	Inbred line
G6	Female	Lx9801	Tangsipingtou	Inbred line
G7	Female	K12	Tangsipingtou	Inbred line
G8	Female	Yuzi87-1	Subtropic group	Inbred line
G9	Female	P138	Subtropic group	Inbred line
G10	Female	Dan598	Lvdahonggu	Inbred line
G11	Female	Zheng22	Lvdahonggu	Inbred line
G12	Female	L217	Lancaster	Inbred line

### Identification of the different kernel types

**Fig. 5:**
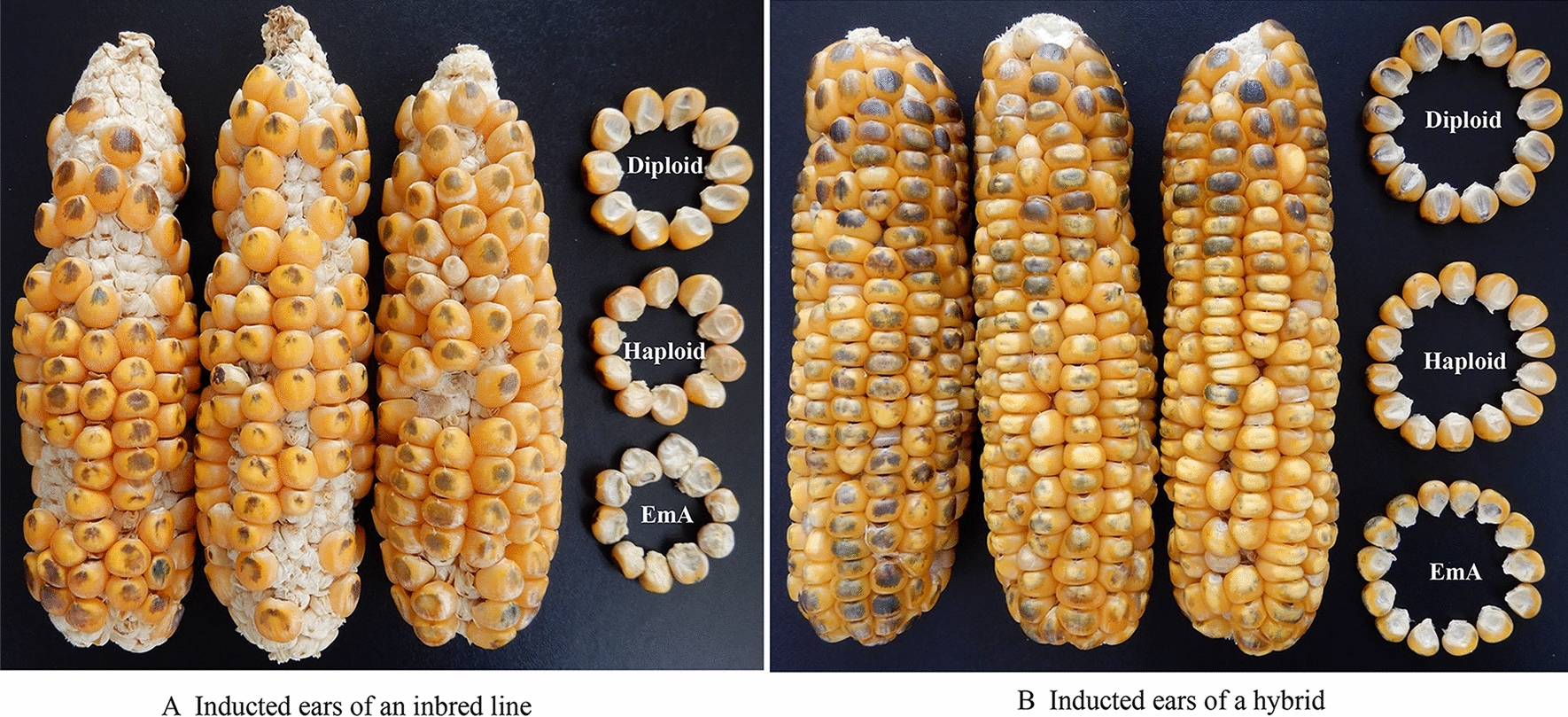
Representative ears and diploid, haploid, and EmA kernels produced during haploid induction. **a** Ears and three kernel types of a maize inbred line pollinated with CHOI2; **b** Ears and three kernel types of a maize hybrid pollinated with CHOI2

The ears of each cross pollinated with inducer lines were harvested at maturity. Haploid kernels were selected based on the phenotype conferred by the dominant marker gene *R1-nj* [9]. Kernels displaying purple endosperm and a purple embryo were classified as diploid, kernels with purple endosperm and a colorless embryo were classified as haploid, and kernels with purple endosperm and no embryo were classified as EmA kernels (Figs. 4,  5). SPSS 19.0 software was used for analysis of the phenotypic data.

$$ {\text{HIR}}\left( \%  \right) = \frac{{{\text{Haploid kernels}}}}{{{\text{Diploid kernels}}{\mkern 1mu} {\text{ + }}{\mkern 1mu} {\text{Haploid kernels}}}} \times \;{\text{100}}\% $$

$$ {\text{EmAR}}\left( \%  \right) = \frac{{{\text{EmA}}}}{{{\text{Diploid  + EmA + }}{\mkern 1mu} {\text{Haploid kernels}}}}\; \times {\text{100}}\% $$

### Kernel sorting system based on a double-threshold for the oil content

**Fig. 6:**
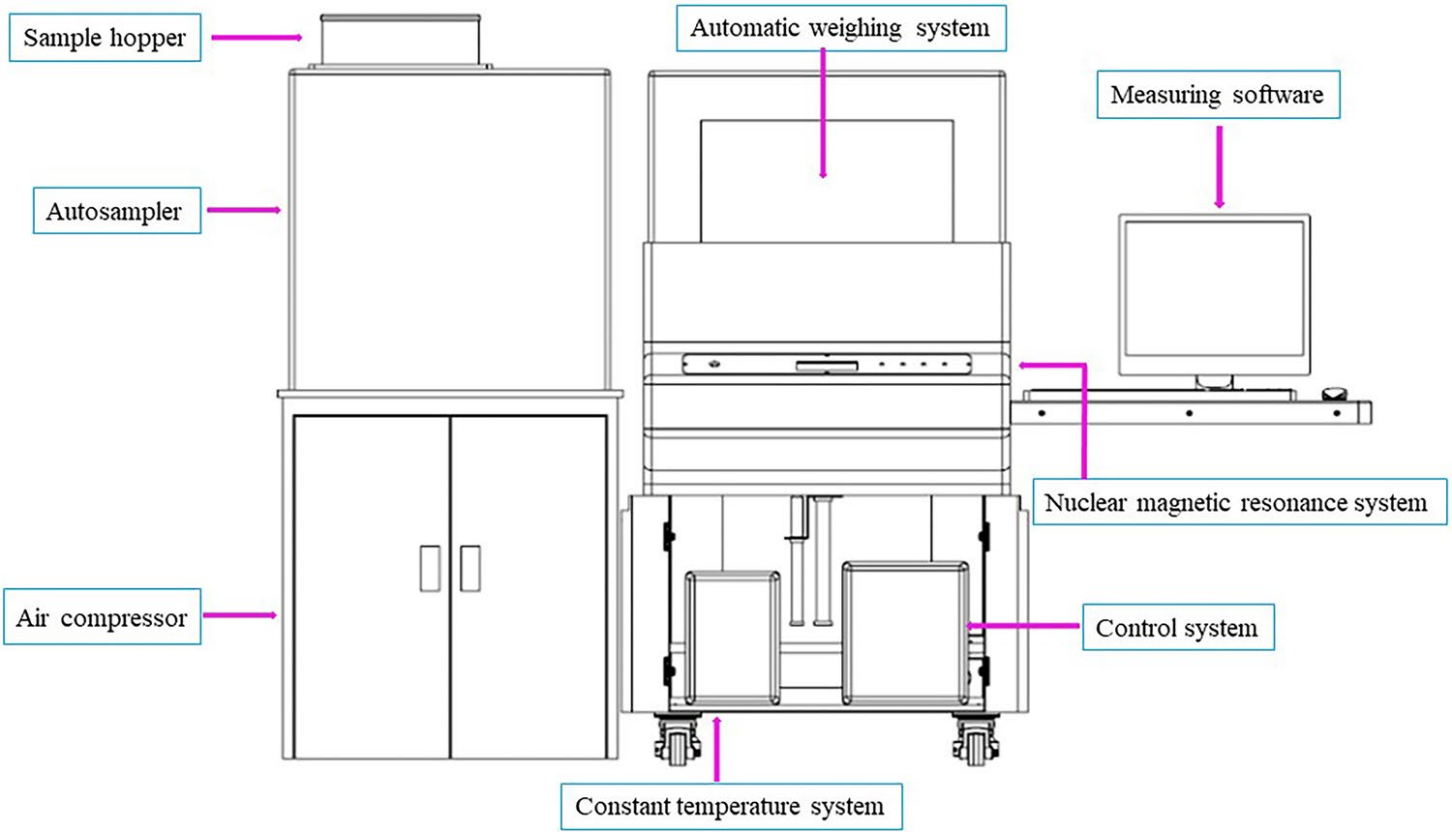
Structural schematic of the NMR sorting system

**Fig. 7:**
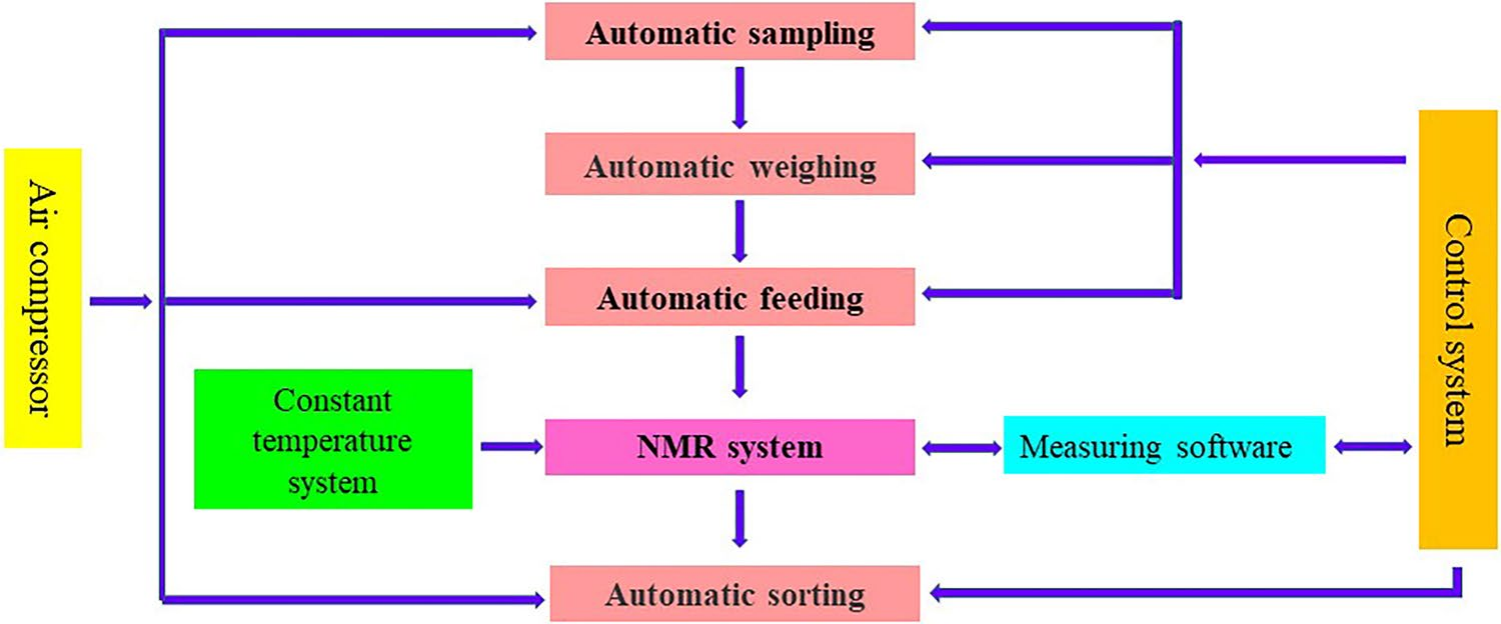
Flow chart of the NMR sorting system

The automated haploid sorting system was based on NMR (model No.: Online MR20-015 V) from Shanghai Niumai Technology Co., Ltd. The equipment mainly consisted of eight parts (Fig. 6): (a) sample hopper, which stored the sample grain to be screened; (b) automatic sampling system, which was composed of a vibration plate and propeller; (c) automatic weighing system, which measured the single kernel weight; (d) air compressor, which provided power for automatic sampling and sorting; (e) constant temperature system, which provided a 35℃ temperature environment for the magnet; (f) a nuclear magnetic resonance (NMR) device, which provided a magnetic field; (g) control system, which controlled the kernel sorting system; and (h) measuring software, which converted the oil component signal into a numerical value, displayed and saved the measured values. The single kernel weight and oil content for diploid, haploid and EmA kernels were measured by the flow chart of the NMR sorting system (Fig. 7). There were three main steps to the operation:

1. Sampling: A vibrating plate was used to transmit kernels, and compressed air pushed them onto the weighing sensor; the weight of the kernel was taken.

2. NMR: Compressed air was used to push the kernel into the sample pipeline and NMR sample holder; NMR measurement was then performed.

3. Sorting: The oil content was determined by a combination of signals for oil based on NMR and the single kernel weight, and the test value of the oil content was compared with the threshold.

**Fig. 8:**
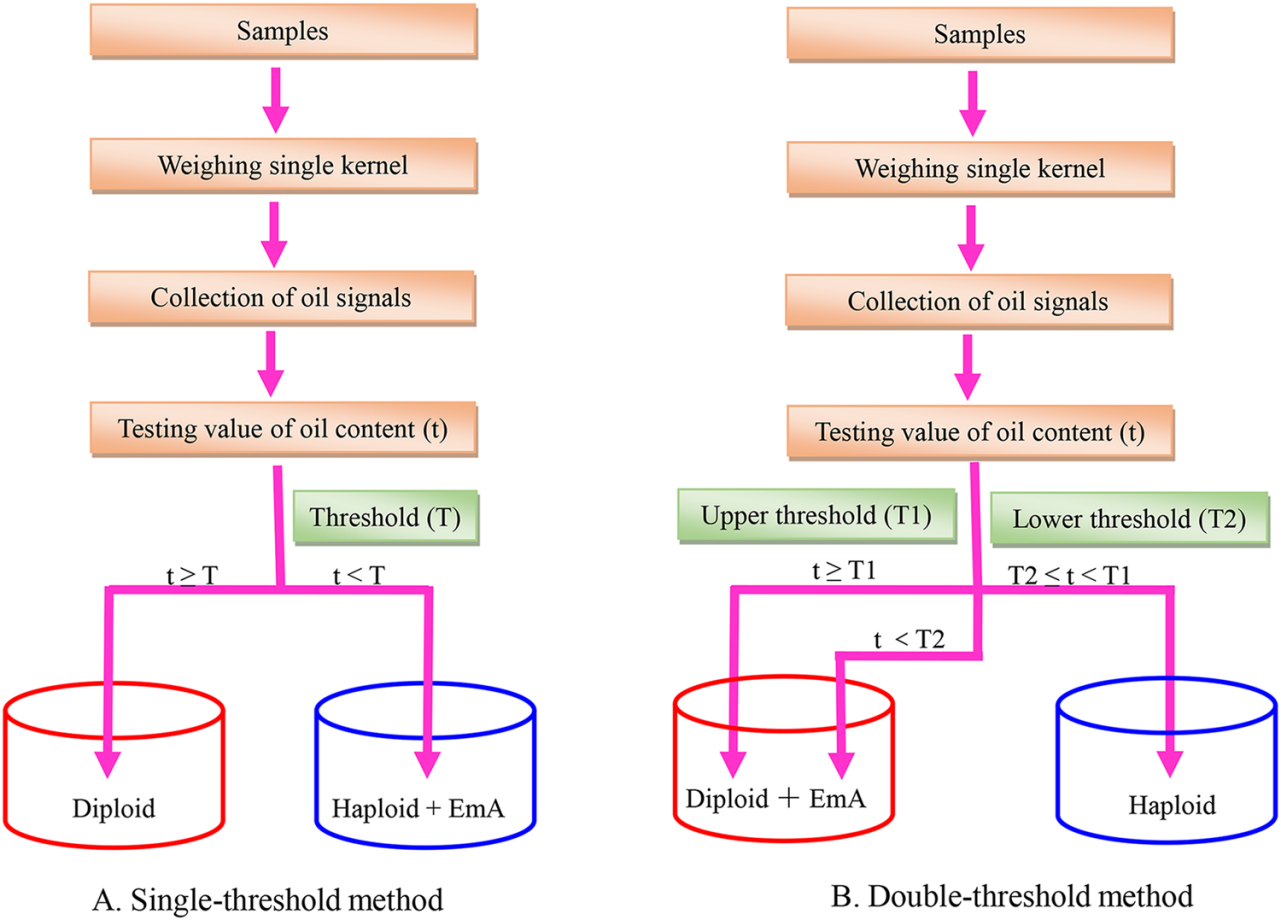
Flow charts showing the maize haploid kernel screening process based on NMR. **a** Single-threshold method, **b** double-threshold method

**Single-threshold:** only one threshold (T1) was set. If the test value (t) of the sample was > T1, the kernel was designated as diploid and sorted into the diploid group. If the test value (t) was < T1, the kernel was considered to be haploid and will be sorted into the non-diploid group (Fig. 8a).

**Double-threshold:** The software set two thresholds, T1 and T2, which were the upper and lower limits of the oil content, respectively. If the test value (t) of the sample was greater than the lower limit value and less than the upper limit value, the sample was considered a haploid kernel and was sorted into the haploid group. If the test value (t) was less than the lower limit value or more than the upper limit, the sample was sorted into the non-haploid group (Fig. 8b).

### Sorting haploid and EmA kernels based on the double-threshold method

Based on the *R1-nj* marker and embryo shape, 150 diploid kernels, 30 haploid kernels, and 20 EmA kernels were selected. The sorting efficiency of EmA kernels was tested using the double-threshold method. A total of 200 kernels were measured five times, and the number of sorted EmA kernels was recorded to calculate the CDR for the sorting of EmA kernels (CDR_*EmA*_) by the following formula:

$${CDR}_{EmA}\left(\%\right)=\frac{{N}_{EmA}}{{T}_{EmA}}\times 100\%$$

***CDR***_***EmA***_, correct discrimination rate of EmA kernels; *N*_*EmA*_*,* the number of EmA kernels in the non-haploid group; *T*_*EA*_, the total number of EmA kernels.

Five oil content thresholds, 5.0%, 4.5%, 4.0%, 3.5%, and 3.0%, were set as the upper limits for the double-threshold method to determine the CDR_H_, CRR_D_ and FDR. The lower limit was set at 2.0%. The number of diploid, haploid, and EmA kernels was recorded to calculate the haploid CDR, FDR, and CRR_D_.

$$ {\text{CDR}}_{{\text{H}}} \left( \%  \right) = \frac{{{\text{N}}_{{\text{H}}} }}{{{\text{T}}_{{\text{H}}} }}\; \times {\text{100}}\% $$

***CDR***_***H***_, correct discrimination rate of haploid kernels; ***N***_***H***_, the number of haploid kernels in the haploid group; ***T***_***H***_, the total number of haploid kernels.

$${\text{FDR}}\left( \%  \right) = \frac{{{\text{N}}_{{\text{D}}} {\text{ + N}}_{{{\text{EmA}}}} }}{{{\text{N}}_{{\text{H}}} {\text{ + N}}_{{\text{D}}} {\text{ + N}}_{{{\text{EmA}}}} }}\; \times {\text{100}}\%  $$

***FDR***, false discrimination rate of sorting; ***N***_***D***_, the number of diploid kernels in the haploid group; ***N***_***EmA***_, the number of EmA kernels in the haploid group; ***N***_***H***_, the number of haploid kernels in the haploid group.

$$ {\text{CRR}}_{{\text{D}}} \left( \%  \right) = \frac{{{\text{N}}_{{\text{D}}} }}{{{\text{T}}_{{\text{D}}} }}\; \times {\text{100}}\%$$

***CRR***_***D***_, correct rejection rate of diploid kernels; ***N***_***D***_, the number of diploid kernels in the non-diploid group; ***T***_***D***_, the total number of diploid kernels.

## Data Availability

The datasets used and/or analyzed during the current study are included within the article.

## Abbreviations

NMR: Nuclear magnetic resonance
CDR: Correct discrimination rate
EmAR: Embryo Abortion Rate
HIR: Haploid Induction Rate
FDR: False discrimination rate
CRR: Correct rejection rate
EmA: Embryo aborted
NIRs: Near-infrared spectroscopy
